# Probing and Manipulating the Lateral Pressure Profile in Lipid Bilayers Using Membrane-Active Peptides—A Solid-State ^19^F NMR Study

**DOI:** 10.3390/ijms23094544

**Published:** 2022-04-20

**Authors:** Stephan L. Grage, Sergii Afonin, Marco Ieronimo, Marina Berditsch, Parvesh Wadhwani, Anne S. Ulrich

**Affiliations:** 1 Karlsruhe Institute of Technology (KIT), Institute of Biological Interfaces IBG-2, P.O. Box 3640, 76021 Karlsruhe, Germany; parvesh.wadhwani@kit.edu (P.W.); anne.ulrich@kit.edu (A.S.U.); 2 Karlsruhe Institute of Technology (KIT), Institute of Organic Chemistry, Fritz-Haber-Weg 6, 76131 Karlsruhe, Germany; marco.ieronimo@outlook.de (M.I.); marina.berditsch@gmx.de (M.B.)

**Keywords:** lateral pressure profile, lipid bilayer, membrane protein, membrane-active amphiphilic peptide, solid-state ^19^F nuclear magnetic resonance, peptide crowding

## Abstract

The lateral pressure profile constitutes an important physical property of lipid bilayers, influencing the binding, insertion, and function of membrane-active peptides, such as antimicrobial peptides. In this study, we demonstrate that the lateral pressure profile can be manipulated using the peptides residing in different regions of the bilayer. A ^19^F-labeled analogue of the amphiphilic peptide PGLa was used to probe the lateral pressure at different depths in the membrane. To evaluate the lateral pressure profile, we measured the orientation of this helical peptide with respect to the membrane using solid-state ^19^F-NMR, which is indicative of its degree of insertion into the bilayer. Using this experimental approach, we observed that the depth of insertion of the probe peptide changed in the presence of additional peptides and, furthermore, correlated with their location in the membrane. In this way, we obtained a tool to manipulate, as well as to probe, the lateral pressure profile in membranes.

## 1. Introduction

The lateral pressure profile has been found to be a key physical property of biomembranes. It plays an important role in the function of membrane proteins, as it can influence their conformation and structure [1,2]. Prominent examples are the mechanosensitive channels, which open in response to a difference in lateral pressure between the two bilayer leaflets, and which can be locked in the open state by the addition of lysolipids to one side of the bilayer [3,4,5,6,7]. The lateral pressure distribution in the membrane is also crucial for the binding and insertion of membrane-active peptides. An important class of such peptides are formed by the cationic amphipathic peptides, which are promising candidates for antibiotics with reduced resistance, due to their ability to bind to and destabilize membranes. Many mechanisms have been discussed to explain their membranolytic activity, which mostly involve partial or full insertion of the peptide into the bilayer [8,9,10]. It has been demonstrated that the insertion of helical antimicrobial peptides depends on the lateral pressure profile of the lipidic environment [11,12]. Whereas lipids with positive spontaneous curvature, i.e., with decreased lateral pressure in the bilayer core, promote insertion of peptides, lipids with negative spontaneous curvature were found to inhibit insertion.

Lateral pressure has been defined by Cantor and others by extending the concept of surface tension to arbitrary layers in the membrane parallel and to the bilayer plane [2,13]. This provides a measure for the lateral forces acting on molecules in the bilayer at different insertion depths. Based on the lateral pressure concept, integral quantities such as the spontaneous curvature can be derived [14,15]. This quantity specifies the curvature that one leaflet of the bilayer on its own would assume, to balance all the lateral forces across the membrane. Both terms are often used synonymously to characterize the “space” available for a guest molecule to insert into the bilayer at a certain insertion depth. However, lateral pressure profile gives a more detailed picture, as it describes the forces as a function of insertion depth. Spontaneous curvature, on the other hand, specifies two curvature radii, and has been related to the second moment of the lateral pressure [16], both of which are integral properties of the lateral pressure profile function. We will, in this account, use the term lateral pressure, as we emphasize the depth dependence of intermolecular forces.

Usually, the lipidic component of biomembranes is considered the major determinant of the lateral pressure profile across the bilayer. Often, lipids are characterized by their spontaneous curvature, or an envelope shape, resulting from the conformational space consumption of the lipid headgroup compared to that of the lipid chains [14,15,16]. For example, lipids with disordered chains, such as unsaturated lipids, or with small headgroups, such as phosphatidylethanolamine, possess a negative spontaneous curvature. On the other hand, lysolipids, for example, cause a positive spontaneous curvature. By choosing an appropriate lipid composition, the lateral pressure profile can be tuned, providing an experimental tool that has been used frequently in the study of peptide or protein interaction with the lipid bilayers [2,3,4,7,11,12].

In this study, we explored the use of peptides instead of lipids to control the lateral pressure profile. In this way, on the one hand, insight into the physical aspects of how membrane-active peptides interact with membranes and might influence membrane constituents, such as membrane proteins, can be gained. On the other hand, peptides could also be a tool for studying the influence of lateral pressure, as they may allow a better tuning of the lateral pressure profile change compared to lipids, due to the versatility of peptide sequence design. Peptides inserting into the interface region of the bilayer have been suggested to modulate the lateral pressure in a similar to lipids possessing a positive spontaneous curvature [17,18,19]. By using a set of peptides (see Figure A3) residing at different insertion depths in the bilayer, we were able to modulate the lateral pressure profile, not only near the bilayer surface, but throughout the entire bilayer. Membrane-inserted peptides were, indeed, found to increase the lateral pressure, specifically at the insertion depth where they resided, through lateral crowding in this layer, and in a similar manner to a lipid with an extended local conformational space. We explored this approach of manipulating the lateral pressure profile through “crowder” peptides, using another peptide as a probe for changes in lateral pressure.

**Figure 1:**
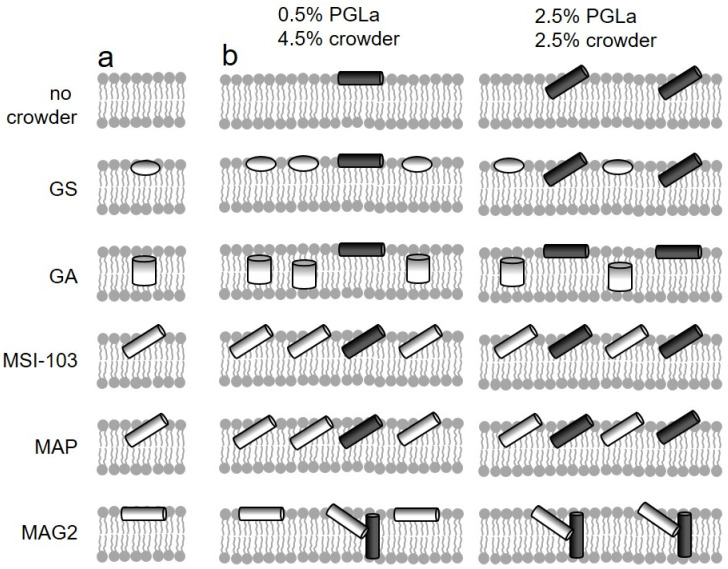
(**a**) Crowding in different regions within the lipid bilayer was created using a series of peptides: the cyclic β-hairpin gramicidin S (GS) was previously found aligned parallel to the surface in DMPC bilayers for most temperatures [31,32], the β-helix gramicidin A (GA) is known to span the bilayer as a dimer [33], the amphiphilic helices of the model peptides MSI-103 and MAP were tilted into the membrane as a T-state at the peptide:lipid molar ratio of this study [36], and the antimicrobial peptide magainin 2 (MAG2), also forming an amphiphilic helix, was found in a surface-parallel or marginally tilted orientation [27,28]. (**b**) In the absence of additional peptides, PGLa assumes an alignment parallel to the membrane surface (S-state) at low peptide:lipid molar ratios (top left) and tilts obliquely into the membrane (T-state) at high peptide:lipid molar ratios (top right). When exposed to these different crowders, PGLa was not influenced by the rather small peptide GS, but responded to crowding by the other peptides in a manner depending on their location in the membrane. PGLa stayed in an S-state in the presence of transmembrane GA, assumed a T-state when mixed with MSI-103 or MAP, which effectively acted like additional PGLa peptide, and inserted in the presence of surface-bound MAG2.

Our experimental strategy is based on distinct states of insertion of peptides into the membrane, ranging from a surface-bound to a membrane-spanning location, which have been characterized for many cationic helical peptide antimicrobials [20]. We were able to read out the insertion state using a solid-state ^19^F-NMR based approach [21,22,23]. In this way, a signature for the peptide response to the membrane environment can be obtained, rendering such peptides as probes suitable for detecting lateral pressure changes. As probes, we used PGLa from the skin of *Xenopus laevis*, a prototype helical antimicrobial peptide (AMP), which was labeled for ^19^F-NMR with 4-(trifluoromethyl)-*L*-phenylglycine (CF_3_-Phg) in position 13, without altering its structure and antimicrobial activity [24,25]. In the fluid phase of the membrane, when PGLa is reconstituted at low peptide:lipid molar ratios, it is aligned parallel to the membrane surface, referred to as “S-state” (see Figure 1b, top). In this alignment, PGLa matches its amphiphilic structure consisting of a hydrophilic and hydrophobic face with the polar/apolar interface of the bilayer. Above a well-defined threshold of the peptide:lipid molar ratio (of 1.25:100 in the case of PGLa), PGLa follows the characteristic behavior of many membrane-active AMPs and tilts into the membrane (“T-state”, see Figure 1b, top). PGLa, finally, completely inserts in an upright orientation (“I-state”) at low temperature, when the bilayer is in the gel phase [26]. Interestingly, this I-state was also induced by another AMP from the same organism, magainin 2 [27,28], with which PGLa forms a synergistically active pair [29,30] (Figure 1b, bottom).

To probe the influence of membrane-inserted peptides on the lateral pressure profile, the realignment of PGLa in the presence of different crowder peptides was evaluated. The crowders were chosen for insertion in different locations in lipid membranes, leading to increased lateral pressure at different depths of the bilayer (Figure 1a and Figure A3). The amphiphilic cyclic β-hairpin gramicidin S (GS), which has been previously found in an S-state alignment in 1,2-dimyristoyl-*sn*-glycero-3-phosphatidylcholine (DMPC) bilayers at most temperatures [31,32], was employed to increase the lateral pressure near the bilayer surface. The hydrophobic β-helix gramicidin A (GA) on the other hand, which spans the membrane as a head-to-head dimer, should increase the lateral pressure in the hydrophobic core of the bilayer [33]. Two further model peptides, MSI-103 and MAP, allowed probing the interaction with peptides of a similar helical and amphiphilic structure, and a similar propensity to tilt into the membrane, as PGLa itself [34,35,36,37]. These two amphiphilic α-helices were observed in a T-state in DMPC bilayers at peptide:lipid molar ratios above 0.42:100 and 0.64:100, respectively, i.e., under all conditions employed in this study [36]. Finally, a further amphiphilic helical peptide, magainin 2 (MAG2), was included in the study, to evaluate the role of specific molecular interactions for the peptide alignment. PGLa and MAG2 exhibited a synergistic antibacterial activity enhancement [29], which is also reflected in their alignment with respect to the membrane. MAG2 possessed a much lower propensity to tilt into the membrane than all the other helical peptides used in this study; however, in the presence of PGLa at a 1:1 molar ratio, it has been found in a T-state orientation [38]. At the same time, PGLa even inserts fully into the membrane in an upright I-state orientation, due the specific interaction with MAG2, and a pore model has been proposed based on these specific intermolecular interactions of PGLa and MAG2 [27,28,38,39].

As a lipid, we chose DMPC, because it exhibits a spontaneous curvature close to zero [40]; and, second, as the behavior in membranes of all peptides used in this study has been described for the case of DMPC. Furthermore, more complex lipid mixtures or variation of lipids would have added a further level of complexity, which we tried to avoid in this biophysical approach.

**Figure A1:**
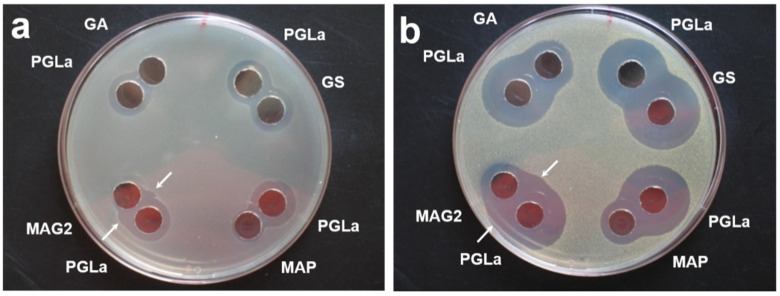
Antimicrobial activity of pairs of the antimicrobial peptide PGLa, labeled with 4-CF_3_-phenylglycine in position 13, with one of the crowder peptides Gramicidin S (GS), Gramicidin A (GA), MAP, or Magainin-2 (MAG2) were examined with respect to their antimicrobial activity against Gram-negative *E. coli* (**a**) and Gram-positive *M. luteus* (**b**), using an agar diffusion assay. Inhibition of growth after incubation of the agar plates at 37 °C overnight is visible as a clear zone around the wells, in which 50 µL of 200 µg/mL peptide solution of the indicated peptide was placed. The synergistically-enhanced activity of PGLa and MAG2 is visible as bulges in the boundary of the inhibition zone (marked by an arrow).

We tested the antimicrobial activity of all peptides paired with the ^19^F-labeled PGLa in agar diffusion experiments, as described in the Appendix A and Figure A1. For all pairs, the known antimicrobial activity was manifested, and PGLa in combination with MAG2 exhibited the expected functional synergy.

To distinguish if the crowder behaves the same as PGLa, i.e., merely leading to an effective increase of peptide concentration, or modulates the insertion behavior of PGLa, two series of samples were employed. In the first series, PGLa was reconstituted into bilayers at a peptide:lipid molar ratio of 0.5:100, below its threshold peptide:lipid molar ratio, and the crowder peptide was added, to result in a total peptide:lipid molar ratio of 5:100, a ratio which lies well above the critical threshold concentration for insertion of PGLa (see Figure 1b, left). In a second series, the PGLa peptide:lipid molar ratio of 2.5:100 was chosen, to be above the threshold ratio for PGLa insertion, and the crowder peptide was added, to result in the same total peptide:lipid molar ratio as in the first series (see Figure 1b, right).

## 2. Results

**Figure 2:**
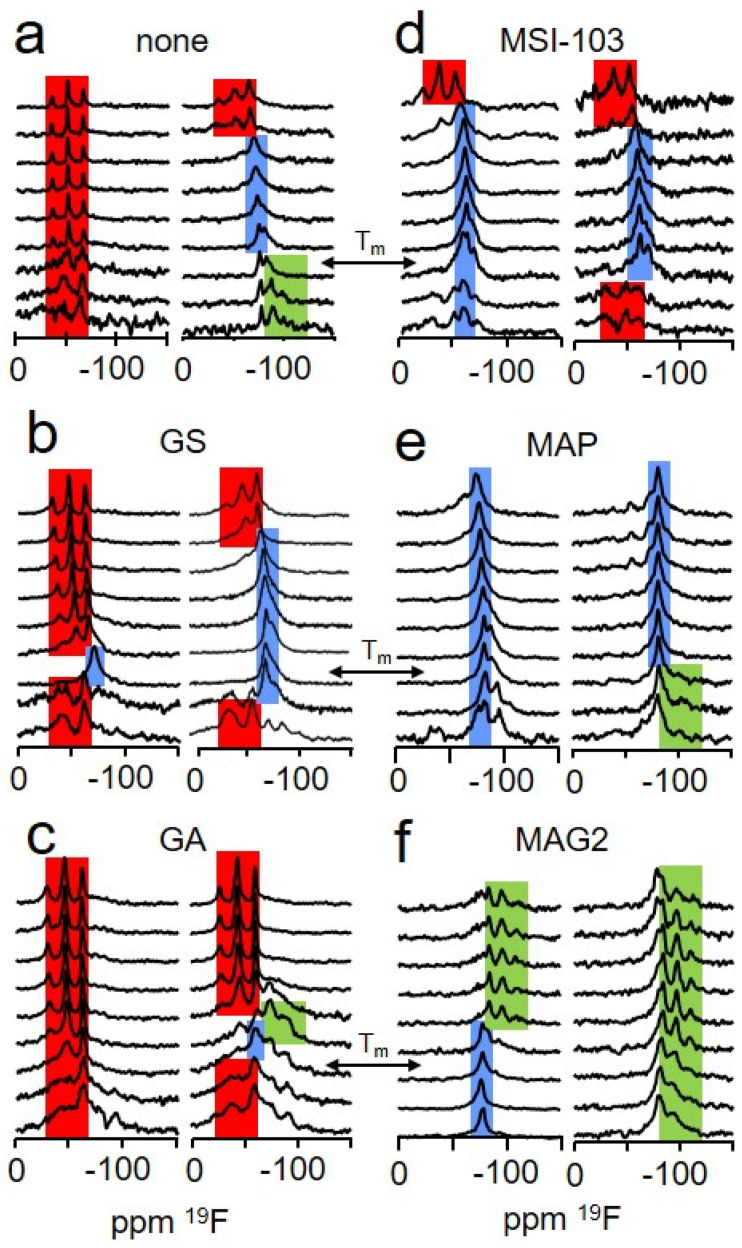
(**a**) The influence of selected crowder peptides (denoted above the spectra), which mimic membrane proteins with different structures and locations, on the orientation of the antimicrobial peptide PGLa, labeled with *CF_3_-Phg* in position 13, was examined on the basis of solid-state ^19^F-NMR spectra. The spectra are indicative of the insertion of PGLa into the membrane in the presence of the crowder. Spectra fingerprints of S-, T-, and I-state are colored in red, blue, and green, respectively. In case of the coexistence of several spectral fingerprints, the pattern is highlighted that caused the highest level of agreement with the respective pattern of the pure PGLa sample in the data analysis. Spectra were acquired of samples at a PGLa:lipid molar ratio of 0.5:100 (left column within each set) and 2.5:100 (right column in each set), and at temperatures from 15 °C (lowest spectrum) to 55 °C, varied in steps of 5 °C. The gel-to-fluid phase transition temperature of DMPC is indicated by “Tm”. A second peptide was added, to result in a total peptide:lipid molar ratio of 5:100. Several crowders were tested: (**a**) reference spectra without crowder, (**b**) with gramicidin S (GS), (**c**) with gramicidin A (GA), (**d**) with MSI-103, (**e**) with MAP, (**f**) with magainin 2 (MAG2).

Experimentally, our analysis was based on solid-state ^19^F-NMR spectra obtained from PGLa, which was reconstituted together with the crowder peptide in mechanically aligned DMPC bilayers [21,23]. To broaden the experimental basis of our conclusions, we measured ^19^F-NMR spectra at a number of different temperatures, from 15 °C to 55 °C. In the obtained ^19^F-NMR spectra (Figure 2), the CF_3_-group of the ^19^F-label gives rise to a triplet signal, which is indicative of the orientation of the helix with respect to the membrane. As the orientation of PGLa in DMPC membranes has been extensively studied previously, in many conditions, including all the peptide:lipid ratios and temperatures used in this study, the ^19^F-NMR signals of the samples containing only PGLa peptide (Figure 2a) could be readily assigned to one of the three known insertion states (S, T, or I) [22,24,25,26,41]. At a low peptide:lipid molar ratio of 0.5:100 (Figure 2a, left column), the ^19^F-NMR spectra display a triplet value and position corresponding to the S-state [24]. At a high peptide:lipid molar ratio of 2.5:100, the triplets correspond to a tilted alignment (T-state) for temperatures above the phase transition (T_m_), and below 50 °C. This concentration-dependent transition from S- to T-state is a typical behavior found for many α-helical amphiphilic peptides [25,36]. Below T_m_, the triplet corresponds to an I-state of PGLa, and above a temperature of ~45 °C to an S-state, as described previously [26].

**Figure 3:**
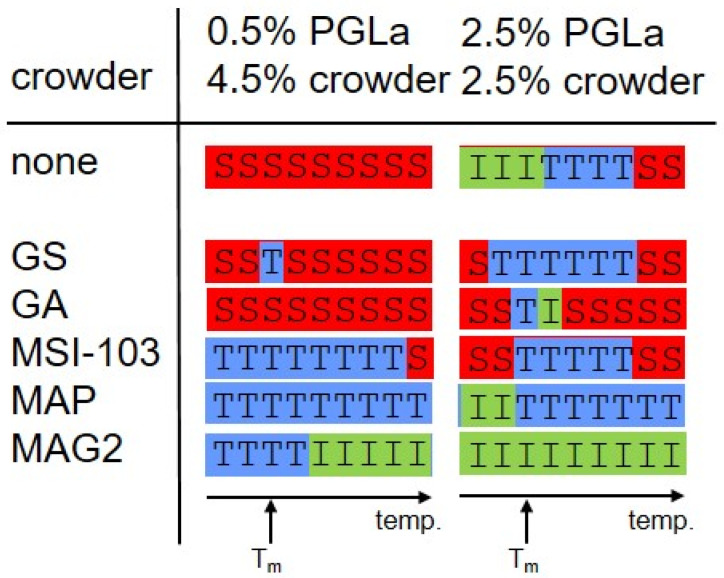
The insertion states of PGLa in DMPC bilayers alone (top) and in the presence of the crowder peptides GS, GA, MSI-103, MAP, or MAG2 were determined from the solid-state ^19^F-NMR spectra of Figure 2, for temperatures from 15 °C to 55 °C, acquired in steps of 5 °C. The spectra were compared with spectra typical for each state (see SI for details), and the state with the best agreement is denoted with S (peptide aligned on the surface, S-state), T (peptide obliquely tilted into the membrane, T-state), or I (peptide inserted in a transmembrane alignment, I-state) for each temperature. Each peptide combination was prepared in two peptide:lipid molar ratios of PGLa:DMPC 0.5:100 and PGLa:DMPC 2.5:100. The crowder peptide was added, to result into a total peptide:lipid molar ratio of 5:100. T_m_ denotes the melting temperature characteristic for DMPC.

**Figure A2:**
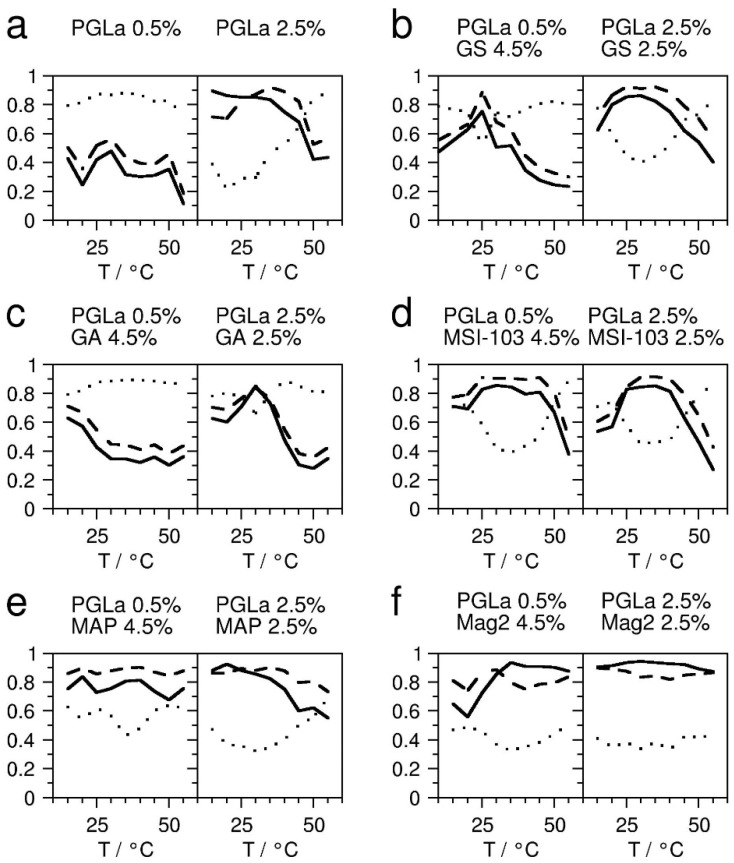
The agreement of the solid-state ^19^F-NMR spectra (see Figure 2) with the characteristic spectrum of one of the respective alignment states of PGLa was quantified using an averaged correlation product, as defined in the text. The averaged correlation products denoting the agreement with an S-state are shown as a dotted line, with a T-state as a dashed line, and with an I-state as a solid line. Samples at a PGLa:lipid ratio of 0.5:100 (left) and 2.5:100 (right) were studied at temperatures from 15 °C to 55 °C, varied in steps of 5 °C. The second crowder peptide was added, resulting into a total peptide:lipid ratio of 5:100. Several crowders were tested: (**a**) reference spectra without crowder, (**b**) with gramicidin S (GS), (**c**) with gramicidin A (GA), (**d**) with MSI-103, (**e**) with MAP, (**f**) with magainin-2 (MAG2).

The insertion states of PGLa in the samples with additional crowder peptides (Figure 2b–f) were assigned by comparison of their ^19^F-NMR spectra with these assigned spectra, using procedures outlined in Appendix B and Figure A2. The insertion states determined in this way are summarized in Figure 3, for both measured peptide:lipid molar ratios and all temperatures.

A very diverse response of PGLa to crowding with the selected peptides was found (Figure 3). GS, for example, exerted almost no influence on PGLa. In the presence of this peptide, PGLa mainly followed the same insertion behavior as a function of temperature as without it. An S-state alignment was observed for the low PGLa concentration sample for nearly all temperatures, similarly to the corresponding sample without GS. In the high PGLa concentration sample the T-state dominated, which changed into the S-state above 45 °C, similarly to the sample without GS. Again, the spectra series resembled that found for the respective samples without GS, with the exception of the dominance of the I-state at low temperatures in the sample containing only PGLa. The experimental findings, hence, are in contrast to expectations, as from the location of GS at the surface of the bilayer (Figure 1a), a realignment of PGLa leading to an enhanced immersion into the membrane would be anticipated.

However, the location of the interacting membrane component within the bilayer did play a role in the case of GA (Figure 3). This crowder peptide prevented the insertion in the form of a T-state or I-state of PGLa, and almost only the S-state was observed for PGLa, at both low and high concentration. This inhibition of insertion by GA can be readily explained by its location in the center of the membrane (Figure 1a), where the β-helix structure of GA seems to be able to increase the lateral pressure in the hydrophobic core sufficiently to preclude PGLa from inserting, and forcing it to stay in the surface-bound S-state.

**Figure A3:**
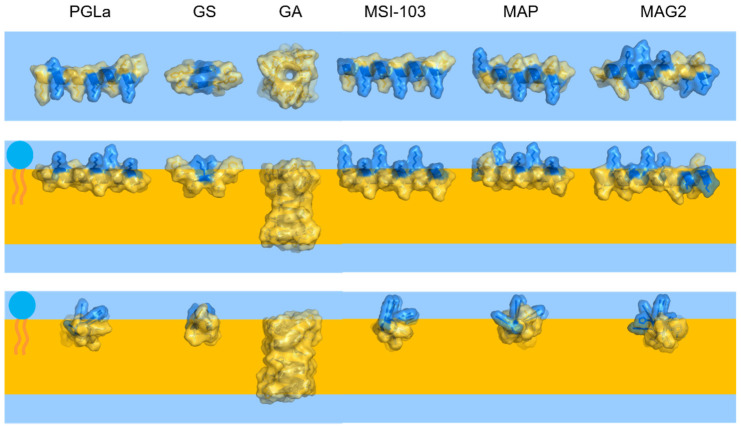
The relative dimensions of the peptides used in this study: PGLa, gramicidin S (GS), gramicidin A (GA, shown as a head-to-head dimer), MSI-103, MAP, and magainin 2 (MAG2). The peptides are shown with their van der Waals interaction surface in top view (top row) and side views (bottom rows). Polar residues are colored in blue, with hydrophobic residues in yellow. The membrane headgroup region and hydrophobic core are indicated by blue and yellow-orange background, respectively.

Hence, the question arises of why GA is able to effect the alignment of PGLa more than GS. At first sight, the different behavior might be due to the smaller size of GS, which has only 60% of the molecular weight of GA. However, mere volume is not decisive for the lateral pressure a peptide or membrane protein exerts on the membrane environment; but rather the variation of size along the membrane spanning direction. For example, a cylindrical object spanning the membrane in its entire length would not change the lateral pressure profile at all, irrespective of its diameter. On the other hand, as the peptides of this study only penetrated into a part of the bilayer, lateral pressure can arise from the fact that the peptide requires space only where it is immersed. In this case, the lateral extension of the peptide would matter for the lateral pressure differences caused by it, and the smaller effect of GS on PGLa might be explained by the slightly smaller cross-sectional area of the cyclic β-hairpin structure of GS, as compared to the β-helix of GA (see Figure A3). Another reason for the stronger effect of GA might lie in its localization in the membrane core, whereas GS is located near the membrane surface. An inhibition of immersion by a peptide in the center of the membrane could, in general, be more easily accomplished than its enforcement by a surfacially-located peptide, for several reasons: an immersion in the headgroup region might be energetically less costly than in the membrane core, due to differences in the lateral pressure profile across the bilayer on its own; other forces such as the interaction of the positive lysine sidechains with the zwitterionic headgroups might keep PGLa in its position near the membrane surface; also the thicker hydrocarbon chain region might provide more room to accommodate active compounds than the thinner headgroup region. MD simulations indeed give hints that the lateral pressure in the headgroup region is lower than in the membrane core [42]. Interestingly, the fact that it is easier to prevent peptide insertion into the bilayer center rather than to cause it has also been observed when looking at the influence of lipids with positive or negative spontaneous curvature on the alignment of α-helical amphipathic peptides, which correspond to crowders near the bilayer surface or in the core of the membrane, respectively. For example, the immersion of MSI-103 is already inhibited by unsaturated lipids with a moderate negative spontaneous curvature, whereas a high percentage of lysolipids with a pronounced positive spontaneous curvature is needed to enforce a transmembrane alignment [11,12].

The two helical amphiphilic model peptides MSI-103 and MAP (Figure 3), on the other hand, predominantly lead to a T-state of PGLa, both below and above its peptide:lipid ratio threshold for insertion. The insertion of PGLa in the presence of these two peptides, thus, resembles that of the highly concentrated sample of pure PGLa, irrespective of the PGLa concentration. Even though the insertion as a function of temperature does not in all details follow that of pure PGLa above the threshold concentration, the most prominent characteristic of the high concentration PGLa state (the T-state above the phase transition temperature) is also dominant with MSI-103 and MAP. These peptides, hence, lead to an effective increase of PGLa concentration, which can be readily explained by their very similar structure and a resulting propensity for membrane insertion. MSI-103 or MAP re-align from S- to T-state above a critical peptide:lipid ratio threshold, similarly to PGLa. From this similarity we also expect the same re-alignment behavior and response to crowding when PGLa is mixed with MSI-103 or MAP. In this way, the cooperative transition from S- to T-state is possible for both pure PGLa and the mixture. A subsequent optimized packing in the T-state may be assisted by structural aspects, which facilitate a dimerization, as discussed previously [25,27]. For example, PGLa possesses a GxxxG motif in the center of the sequence, and in the design of MSI-103, three glycines have been included, in such a way that they are on the same side of the helix, facilitating helix–helix interactions in both cases [12,37]. However, we also note that addition of neither PGLa-like peptides leads to a notable synergistic activity enhancement.

MAG2, although possessing a similar helical amphiphilic structure to MSI-103 or MAP, modulated the insertion behavior of PGLa in a very different way (Figure 3). With MAG2, PGLa was found to be inserted in an I-state, both at low and high PGLa concentrations for most temperatures. Our experiments, hence, confirm previous observations of a transmembrane alignment of PGLa in the presence of MAG2 [27,28,38,39,43]. The influence of MAG2 on PGLa can be interpreted as a response to lateral crowding of a crowder in the polar/apolar interface region of the membrane and subsequent change in the lateral pressure profile. Differently from MSI-103 or MAP, MAG2 has previously been shown to possess a high propensity to adopt an S-state [27,28]. This preference for the interface region of the bilayer may increase the lateral pressure profile in the headgroup region, and, hence, force PGLa to insert into the membrane in an upright orientation at a high overall peptide density. On the other hand, it has been recently found that MAG2 and PGLa interact at a molecular level; likely via a GxxxG motif on PGLa, or involving the negatively charged E19 residue of MAG2, giving rise to oligomeric structures, where MAG2 assumes a tilted orientation [38,39]. Hence, the observed response of PGLa to the addition of MAG2 can be well explained by the formation of supramolecular structures mediated by specific intermolecular interactions. It may also be conceivable that both, a lateral pressure increase near the bilayer interfaces induced by MAG2 and a specific interaction act together, where a re-alignment due to crowding turns MAG2 and PGLa into a position favorable for an inter-molecular interaction.

## 3. Discussion

Our results reveal that, besides specific intermolecular interactions, a purely physical mechanism can also cause peptide–peptide interactions in membranes. Similarly to lipids, we found that peptides can also cause a change of the lateral pressure profile in the bilayer through a locally high peptide density (see Figure 1b). In this way, in our experiments, crowder peptides were able to alter the orientation of the PGLa, which was used as a probe. Crowders in the center of the bilayer were found to exclude PGLa from the membrane interior, whereas crowders bound to the membrane surface promoted the insertion of PGLa. With crowders of equal structure and location in the membrane, PGLa adopted the same obliquely tilted alignment as the crowder itself, which achieved optimal spatial packing at generally increased lateral pressure. Hence, a coherent picture emerged, whereby, on the one hand, membrane-inserted peptides as active players increase the lateral pressure due to their presence. On the other hand, peptides also respond to the lateral pressure changes as passive players, by avoiding membrane regions of increased lateral pressure. Often, either the active or the passive role in modifying the lateral pressure profile prevails. For example, curvature sensing peptides, such as epsin [44], can, by actively generating curvature, facilitate membrane insertion of cell penetrating peptides, whereas many antimicrobial peptides, such as the here-described PGLa, respond more passively to the membrane environment. Hence, not only lipids, but also membrane-binding or -inserting peptides provide a versatile tool for manipulating the lateral pressure profile. Moreover, peptides, with their wide spectrum of membrane interactions and locations within the membrane, allow targeting the lateral pressure in any region throughout the bilayer, with fewer restrictions than lipids with different spontaneous curvature. Equally, peptides can serve as probes for lateral pressure, by translating lateral pressure into a detectable quantity, such as a helix tilt angle. However, membrane-inserting peptides not only present experimental tools for gaining insight into intermolecular forces and the physical properties of membranes, the interplay between manipulation and sensing lateral pressure may be considered as a model of intermolecular interactions in biomembranes. Membrane proteins constitute a major part of biomembranes, with mass percentages of above 50%, and should have an equal influence on membrane-active peptides as the crowders in this study had on the probe peptide. Hence, for example, for the activity of membrane-active antimicrobial peptides, membrane proteins may play an equally important role as the lipid components. However, whereas the dependence of antimicrobial activity on the physical properties of lipid bilayers has been studied intensively, our study provides a new perspective on the importance of the proteinous part of biomembranes for the activity of membrane-targeting agents. To obtain an insight into a particular aspect of the peptide interplay with membranes, namely the effects involving changes in the lateral pressure profile, it was necessary to use model membranes in this study, to reduce the complexity inherent in natural membranes. However, as the lateral pressure is a mere physical effect, it will also be present in native membranes; and the results of this study should be transferable to natural membranes. It remains an important question for future studies, to evaluate how large the contribution of such lateral pressure related effects are within the concert of other interactions and processes present in natural membranes.

## 4. Materials and Methods

*Peptide synthesis and sample preparation*. Gramicidin S (cyclo-[fPVOL]_2_, with f = *D*-phenylalanin, O = ornithine) was obtained by fermentation of the soil bacterium *Aneurinibacillus migulanus,* as described previously [45]; and gramicidin A (formyl-XGAlAvVvWlWlWlW-ethanolamine, X = Val or Ile, lower-case signifies *D*-enantiomer) was obtained from Sigma-Aldrich. The peptides PGLa (GMASKAGAIAGKXAKVALKAL-carboxyamide), MSI-103 ([KIAGKIA]_3_), MAP (KLALKLALKALKAALKLA), magainin 2 (GIGKFLHSAKKFGKAFVGEIMNS) were synthesized using standard Fmoc solid phase synthesis [46,47], following procedures described previously [25,26,48]. All peptides except gramicidin A were purified by reverse phase HPLC, employing a C18 column and acetonitrile:water gradients. PGLa was fluorine-labeled by introducing *D*/*L* 4-trifluoromethyl-phenylglycine (ABCR, Karlsruhe, Germany) in position 13 (leucine in the original peptide, marked with X in the above sequence), and separating the diastereomeric mixture by HPLC. 

For oriented samples, 0.3–0.5 mg of labeled PGLa, one of the other peptides and dimyristoyl-phosphatidylcholine (Avanti Polar Lipids, Alabaster, AL, USA) were co-dissolved in a 1:1 (*vol*:*vol*) chloroform/methanol, with amounts adjusted to yield the desired molar ratios. The solution of each sample was spread on 10–15 glass slides (7.5 × 18 × 0.06 mm, Marienfeld, Germany), which were dried under vacuum, stacked, and covered with an empty glass slide. The stacks were incubated at 97% relative humidity and 48 °C for 24 h to hydrate the bilayers, and sealed in parafilm and clinch foil. This sample preparation procedure led to stacks of ~10^3^ hydrated bilayers with reconstituted peptides. Successful reconstitution following this protocol has been demonstrated in numerous studies, e.g., for the alignment of antimicrobial peptides [11,12,24,25,26,31,32,43]. In the case of the a-helical peptides, correct folding when associated to membranes has been previously validated using circular dichroism [36]. 

*NMR spectroscopy*. Solid-state ^19^F-NMR experiments were carried out on a 500-MHz Bruker spectrometer (Avance console) operating at 470.7 MHz ^19^F resonance frequency, using a double resonance ^19^F/^1^H probe equipped with a turnable flat coil resonator, to fit the oriented samples from Doty Scientific (Columbia, SC, USA). For each spectrum, approximately 2000 scans of 10 ms free induction decay under ~15 kHz ^1^H-decoupling with a recycle delay of 2 s were acquired.

## Data Availability

The data presented in this study are shown in the figures of this article. Raw data are available from the corresponding authors on request.

## Appendix A. Antimicrobial Activity

We examined the antimicrobial activity of PGLa, labeled with 4-CF_3_-phenylglycine in position 13, in combination with each crowder peptide, using an agar diffusion assay. The activity against *Escherichia coli* DSM 6897 and *Micrococcus luteus* DSM 20030 was tested, as representatives of Gram-negative and Gram-positive bacteria, respectively. The agar plates for the agar diffusion assay were prepared as follows: Aliquots of bacterial culture were taken during the exponential growth phase, and were diluted to a suspension with an OD^550^ of 0.2. For inoculation, 50 µL or 100 µL of these bacterial suspensions, respectively, were added into 17 mL aliquot of melted “no salt” Luria agar that had previously been incubated at 60 °C. This mixture was poured into a Petri dish. After the solidification of the agar medium, two wells of 9 mm diameter were punched in the agar plate for each peptide pair, and 50 µL of a 200 µg/mL solution of the desired peptides were added into the wells. The agar plates were incubated at 37 °C overnight and growth inhibition was evaluated visually as clear zones around the wells.

All tested peptides exhibited antimicrobial activity against both strains, except for GA, which was only active against Gram-positive *M. luteus* (Figure A1). (The activity of MAP against *E. coli* was lower than that of the other peptides, and the inhibition zone in the case of MAG2 was distorted, nonetheless both peptides showed activity). However, only in the case of the pair of PGLa and MAG2 was this activity synergistically enhanced, as visible from the bulges in the boundary of the inhibition zone (see arrow in Figure A1).

## Appendix B. Determination of the Peptide Insertion States

The dominant alignment states of PGLa, reconstituted together with a second crowder peptide into oriented DMPC bilayers, were obtained from the solid-state ^19^F-NMR spectra of the ^19^F-label introduced into PGLa (see manuscript Figure 1). The basis for this determination from a single ^19^F-NMR spectrum was the previously detailed solid-state NMR studies of the orientation of PGLa in DMPC membranes under a wide range of temperatures and peptide:lipid ratios, which employed at least four labeled positions [25,26,41]. From these studies, the insertion states of PGLa alone, without a crowder peptide, were known at the temperatures and peptide:lipid ratios used in this study. The spectra of the low PGLa concentration sample with a peptide:lipid ratio of 0.5:100 could in this way be assigned to an S-state (spectra in manuscript Figure 2a, left column). The spectra of the high PGLa concentration sample of a peptide:lipid ratio of 2.5:100 were assigned to I-state in the temperature range 15–25 °C, to T-state in the range of 30–45 °C, and to S-state in the range of 50–55 °C (spectra in manuscript Figure 2a, right column). To determine the insertion state of the mixed samples containing two peptide species, their ^19^F-NMR spectra (Figure 2b–f) were compared with each spectrum of the set of spectra from samples with only PGLa in DMPC with the known assignment described above (Figure 2a). The agreement of these pairs of spectra was quantified using a correlation product *P*, defined as follows:

*P* = ∑*_i_* (*A_i_ B_i_*)/[ ∑*_i_* (*A_i_ A_i_*) ∑*_i_* (*B_i_ B_i_*) ]^1/2^ (1)

where *A_i_* and *B_i_* denote the amplitudes at the *i*th point of the two correlated spectra, which were normalized to ∑*_i_ A_i_* = 1 and ∑*_i_ B_i_* = 1, and corrected for baseline offset.

All correlation products comparing a particular spectrum with an assigned spectrum of the same insertion state (either S, T or I) were averaged. These averaged correlation products were used as a measure of the agreement of the particular spectrum with the respective state. The averaged correlation product can assume values between 0 and 1, and increases with increasing match. Figure A2 summarizes the obtained agreement of each sample, and at each temperature, with the S-state, T-state, and I-state.
